# Application of Infrared and Raman Spectroscopy for the Identification of Disease Resistant Trees

**DOI:** 10.3389/fpls.2015.01152

**Published:** 2016-01-07

**Authors:** Anna O. Conrad, Pierluigi Bonello

**Affiliations:** ^1^Forest Health Research and Education Center, Department of Forestry, University of KentuckyLexington, KY, USA; ^2^Department of Plant Pathology, The Ohio State UniversityColumbus, OH, USA

**Keywords:** trees, disease, resistance, infrared and Raman spectroscopy, chemometrics

## Abstract

New approaches for identifying disease resistant trees are needed as the incidence of diseases caused by non-native and invasive pathogens increases. These approaches must be rapid, reliable, cost-effective, and should have the potential to be adapted for high-throughput screening or phenotyping. Within the context of trees and tree diseases, we summarize vibrational spectroscopic and chemometric methods that have been used to distinguish between groups of trees which vary in disease susceptibility or other important characteristics based on chemical fingerprint data. We also provide specific examples from the literature of where these approaches have been used successfully. Finally, we discuss future application of these approaches for wide-scale screening and phenotyping efforts aimed at identifying disease resistant trees and managing forest diseases.

## Introduction

Forest tree species are increasingly being threatened by native and introduced pathogens as a result of globalization and climate change, which can alter the life cycles of pathogens that have co-evolved with tree hosts and facilitate the spread of non-native and invasive pathogens to areas where native tree species lack specific defenses against them. Because non-native and invasive species continue to be introduced, regulations have been imposed by government organizations to curtail the spread of pathogens capable of causing disease in naïve habitats (Frankel, 2008; Potter et al., 2011). However, these efforts have had no real impact on our ability to contain pathogens due to failure to sever the pathways by which non-native and invasive species enter naïve areas (Liebhold et al., 2012), uncontrollable pathogen dispersal patterns and potential, and the effects of global change, which has made many areas more suitable to invasion by destructive agents (Rustad et al., 2012; reviewed in Sturrock et al., 2011).

In general, few options exist for managing forest diseases, with preventative management (e.g., pathogen exclusion) likely to be the most effective. Other options such as chemical control, either before or after infection, may be effective on individual trees, e.g., in the urban landscape, but are logistically and economically impossible, and environmentally unacceptable, on large spatial scales, e.g., in a forest stand or at a landscape level. For these reasons, the identification and utilization of resistant trees for disease management and restoration of disturbed habitats should be a top priority (Telford et al., 2015; Wingfield et al., 2015). In many instances, intensive artificial selection of resistant trees is required since there is an insufficient amount of quantitative genetic variation to allow for natural recovery in forest populations (Ennos, 2015). Examples of where resistant trees have been identified and bred using intensive artificial selection include: inter-specific chestnut hybrids resistant to *Cryphonectria parasitica* (causal agent of chestnut blight) (Burnham, 1988), Port-Orford-cedar resistant to *Phytophthora lateralis* (causal agent of Port-Orford-cedar root rot) (Sniezko et al., 2012), and elm tolerant to *Ophiostoma novo-ulmi* (causal agent of Dutch elm disease) (Martín et al., 2015).

However, even successful breeding programs face many challenges, including dealing with long lifespans and generation times of most tree species (Telford et al., 2015). For example, breeding programs for chestnut, Port-Orford-cedar, and elm have taken decades to produce any resistant or tolerant germplasm (Burnham, 1988; Oh et al., 2006; Martín et al., 2015). In addition, methods for screening and phenotyping trees for disease **resistance** typically rely on artificial inoculation or natural infection of individual trees. This approach is labor and time intensive, and is not suitable for rapid, high-throughput screening, or phenotyping (Neale and Kremer, 2011). Finally, in instances where intensive artificial selection is not feasible, high-throughput methods, not reliant on artificial or natural infection, are needed to assess the proportion of resistant trees within a population. This type of information can be used to facilitate and dictate what, if any management practices are implemented.

KEY CONCEPT 1. **Resistance**

Trees are classified as resistant based on qualitative and/or quantitative attributes. For example, a tree with a lesion length, caused by pathogen infection, at or below some critical threshold, could be defined as resistant. A tree that no longer shows active symptoms of disease, after an initial display of those symptoms, i.e., a tree that is in remission, could also be considered resistant.

Genetic and genomic approaches, including genomic selection for quantitative traits, like disease resistance, are being developed and are currently used in some high value tree crops such as *Pinus taeda* (loblolly pine) (Resende et al., 2012). However, these approaches have not been widely implemented because the genetic basis of host resistance is still unknown for many forest pathosystems (Neale and Kremer, 2011; Muranty et al., 2014). Therefore, more rapid and less expensive alternative approaches are needed for screening and phenotyping trees, especially non-model tree species.

One such approach, which has the potential to overcome current screening and phenotyping limitations, utilizes infrared (IR) or Raman spectroscopy to generate chemical fingerprints of biological samples. **Chemical fingerprinting** can be combined with **chemometrics**—multivariate analysis of chemical data—to identify spectral features that differentiate two or more groups (Fiehn, 2001, 2002; Goodacre et al., 2004; Xia and Wishart, 2011).

KEY CONCEPT 2. **Chemical fingerprinting**

A comprehensive analysis of all the chemicals (metabolites) present within a given sample; individual chemicals are not separated, identified, or quantified.

KEY CONCEPT 3. **Chemometrics**

Multivariate statistical analysis of chemical data that is aimed at identifying differences between two or more groups. In the context of tree disease resistance, chemometrics is used to identify spectral signatures that are capable of distinguishing between resistant and susceptible trees.

Vibrational spectroscopy-based approaches, like Fourier-transform mid-infrared (FT-IR) spectroscopy, near-infrared (NIR) reflectance spectroscopy, and Raman spectroscopy typically are more rapid, reproducible, and reliable than traditional metabolomic methods, like high performance liquid chromatography (Fiehn, 2001, 2002). Chemical fingerprint data generated from IR and Raman spectrometers can be analyzed using various chemometric methods, like principal components analysis (PCA), soft independent modeling of class analogy (SIMCA), or partial least squares regression (PLSR) (Goodacre et al., 2004; Allwood et al., 2008; Cozzolino, 2014).

Although spectroscopic approaches have been widely used to assess the physiological status of trees, particularly in relation to water stress, there are relatively few examples of where vibrational spectroscopy-based methods, combined with chemometrics, have been used to distinguish between trees that vary in disease susceptibility. Early examples are provided in a series of papers by Martin et al. in the mid to late 2000s, in which the authors detailed the use of FT-IR spectroscopy, combined with chemometrics, to distinguish between elm species and clones that differed in susceptibility to *O. novo-ulmi* before and after infection (Martin et al., 2005a,b; Martín et al., 2007, 2008). More recently Conrad et al. (2014) utilized FT-IR spectroscopy combined with chemometrics to identify *Quercus agrifolia* (coast live oak) naturally resistant to the non-native and invasive pathogen *Phytophthora ramorum*, the causal agent of sudden oak death.

The extensive use of vibrational spectroscopy-based methods in many different scientific disciplines is a testament to their general applicability and relative ease of use. For example, these methods have been used for human disease diagnosis (Ellis and Goodacre, 2006) and for general plant phenotyping (Cozzolino, 2014; Li et al., 2014). However, these methods have not been widely adopted for the study and identification of disease resistant trees, perhaps because most forest pathologists, and tree breeders are unaware of their potential applicability to this field. Therefore, the focus of this review is to: (1) summarize commonly used vibrational spectroscopic tools and chemometric methods, (2) provide specific examples of where such tools have been used in the context of trees and tree diseases, and (3) discuss future applications of this technology for large-scale screening and identification of disease resistant trees.

## Vibrational spectroscopy-based methods for chemical fingerprinting

Vibrational spectroscopy-based methods, which include IR and Raman spectroscopy, can produce chemical fingerprints of solid, liquid, and gaseous samples, and have the capability to be adapted for high-throughput analysis (Diem, 1993; Fiehn, 2001). Solid samples include intact leaf or twig tissue, or ground tissue. Liquid extracts from tree tissues can also be analyzed, often with benchtop or portable (i.e., smaller and easier to transport) devices in laboratory settings. Handheld devices can be used beyond the laboratory and are most appropriate for in-field or forest applications.

### Fourier-transform (mid)-infrared (FT-IR) spectroscopy

FT-IR spectroscopy measures changes in the molecular absorption of IR radiation and vibrations (e.g., stretching, bending, deformation), which are influenced by molecular structure (Diem, 1993; Guillén and Cabo, 1997; Ellis and Goodacre, 2006; reviewed in Rodriguez-Saona and Allendorf, 2011). It is commonly used to examine the mid-infrared (MIR) spectral region, which ranges from 4000 to 600 cm^−1^ (cm^−1^—inverse wavenumbers) or 2500–25,000 nm (reviewed in Ellis and Goodacre, 2006; Cozzolino, 2014). FT-based methods use interferometers to collect and focus emitted light, and with FT-IR spectroscopy all the wavelengths are analyzed simultaneously as they arrive at the detector (Diem, 1993).

**Figure 1:**
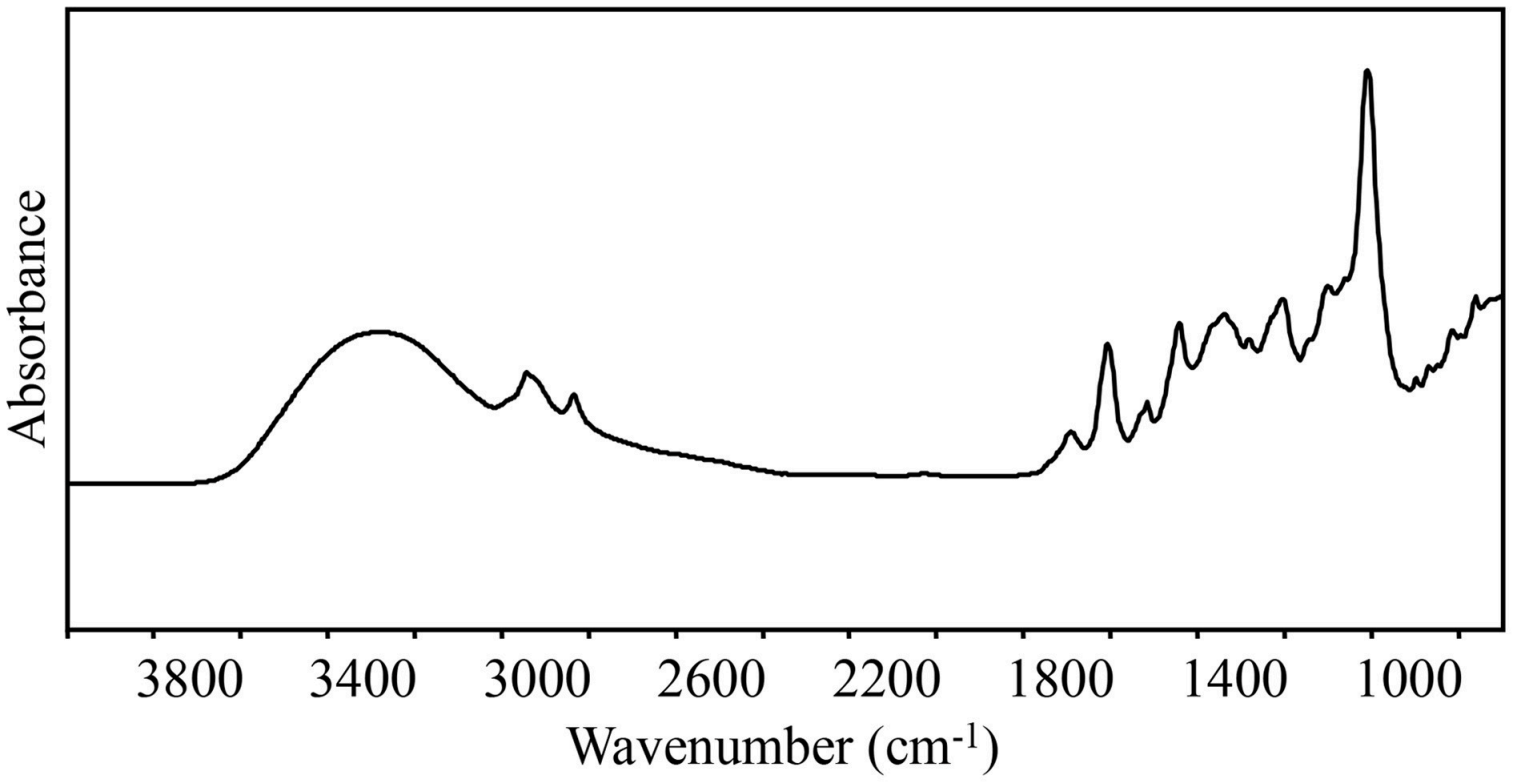
**A representative chemical fingerprint using raw spectra collected in the mid-IR region (4000–700 cm^**−1**^) from ***Quercus agrifolia*****. FT-IR spectroscopy produces chemical fingerprints that can be analyzed using chemometrics to identify spectral differences between resistant and susceptible trees.

Fourier transformation is also combined with NIR and Raman spectroscopy; however, for the purpose of this review, FT-IR spectroscopy will be used to describe analyses of the MIR spectral region, unless otherwise noted. The MIR spectral region contains many sharp peaks and thus is information rich (Figure 1), which is why it is often said to contain the fingerprint region or zone (Ellis and Goodacre, 2006). One potential disadvantage of using the MIR spectral region for analysis is that water absorbs strongly in this region. To mitigate this, different approaches can be used to remove potentially obscuring signals, such as sample dehydration or utilization of attenuated total reflectance attachments (reviewed in Ellis and Goodacre, 2006).

Laboratory benchtop and portable FT-IR spectrometers are most commonly used for sample analysis, although handheld devices equipped with attenuated total reflectance accessories are available (Sorak et al., 2012; Santos et al., 2013). Handheld devices could be readily adopted for in-field (forest) use, though device size may impact measurements, since smaller devices may have reduced performance (Sorak et al., 2012). Still, the obvious benefits of handheld devices—i.e., potential for in-field measurement of chemical fingerprints, without the need to process samples in the laboratory—would likely outweigh any reduction in measurement performance.

### Near-infrared (NIR) spectroscopy

Like with FT-IR spectroscopy, the attenuation of the IR beam as it passes through the sample is monitored by NIR spectroscopy as a function of wavelength (or wavenumber) (Diem, 1993). Minimum sample preparation is required for NIR spectroscopy, and sample analysis is relatively straightforward. NIR spectrometers—which cover spectral ranges from 13,400 to 4000 cm^−1^ (750–2500 nm)—are capable of analyzing organic chemical structures containing O-H, N-H, and C-H bonds (Foley et al., 1998; Cozzolino, 2014). Unique physico-chemical properties of different molecules result in characteristic spectra through wavelength-dependent scattering and absorption (Cozzolino, 2014). However, unlike analysis in the MIR region, peaks from NIR spectroscopy are not distinct or sharp and can be lower in intensity (Foley et al., 1998; Cozzolino, 2014).

There are many commercial options available for high-throughput screening and/or phenotyping using portable and handheld NIR spectrometers, some of which come equipped with interchangeable accessories, like fiber-optic probes (Foley et al., 1998; Sorak et al., 2012; Warburton et al., 2014). While NIR spectrometers may be less sensitive and accurate than FT-IR spectrometers and Raman spectrometers (see below), ease of use and generally lower costs for NIR sample analysis make it an ideal tool for rapidly screening trees for disease resistance (Lupoi et al., 2014).

### Raman spectroscopy

Raman spectroscopy also is capable of producing chemical fingerprints; however, Raman spectroscopy measures the exchange of energy at a given wavelength after molecules are irradiated with an excitation source, such as a laser (Ellis and Goodacre, 2006; Lupoi et al., 2014). Instead of measuring the attenuation of light itself (as in IR spectroscopy), Raman spectroscopy measures the spectra of scattering photons coming from the sample (Diem, 1993). Raman shifts in wavelength of incident laser light are analogous to IR absorption by molecules after they are interrogated with an IR beam (Ellis and Goodacre, 2006). For this reason, data analysis is essentially the same for IR and Raman spectroscopy.

One potential disadvantage of using Raman spectroscopy is that the Raman effect may be weak, requiring longer signal collection times (i.e., the amount of time spectra must be collected by the detector) (Ellis and Goodacre, 2006). Still, there are many advantages to using Raman spectroscopy. For example, handheld and portable Raman spectrometers may be more appropriate for on-site qualitative analysis than other IR-based devices, because the laser focus can be positioned directly on a sample—e.g., a sample can be analyzed even if stored in a plastic bag or glass vial—and water does not have an obscuring effect on spectra (Ellis and Goodacre, 2006; Sorak et al., 2012).

## Chemometric analysis of chemical fingerprint data

Regardless of the approach used to generate chemical fingerprints of biological samples, fingerprint data must be analyzed using appropriate methods (i.e., chemometrics) in order to reveal underlying spectral patterns that may be associated with variation between groups, either before or after pathogen infection, and ultimately to develop accurate models for predicting tree resistance. There are three primary steps to analyzing chemical fingerprints: (1) pre-processing of raw data to minimize background noise and to improve model fit; (2) chemometric methods, including methods for data visualization, data mining, data reduction, and predictive modeling; and (3) model validation, including methods for assessing model fit and accuracy.

### Data pre-processing

Pre-processing methods are needed to deconvolute overlapping bands (peaks), minimize background and noise, and improve model predictions. There are a variety of different methods for spectral data normalization and smoothing, which can be used individually or in combination. Commonly used methods for pre-processing spectral data can be found in Table 1.

**Table 1:** **Commonly used pre-processing methods for infrared spectroscopy and Raman spectroscopy derived data**.

**Pre-processing method**	**Description**
Standard normal variate (SNV)	Multiplicative scatter and particle size interference is removed (Barnes et al., 1989).
Multiplicative scatter correlation (MSC)	Corrects for noise and scatter; removes multiplicative effects (Lupoi et al., 2014).
Derivative	First and second derivative functions are commonly used to reduce error and resolve overlapping bands (peaks) (Sankaran et al., 2010).
Savitzky-Golay polynomial filter	For smoothing and derivatizing data (Gierlinger et al., 2003).
Detrending	Corrects for variation in baseline shifts and co-linearity (Barnes et al., 1989).

### Chemometric methods

In order to use chemical fingerprints to develop models capable of accurately grouping trees based on variation in susceptibility, data need to be visualized and the most important spectra for discriminating between groups need to be identified (Gierlinger et al., 2003; Durgante et al., 2013). The chemometric methods used should be able to reduce the dimensionality of data and handle potential co-linearity between variables. These methods should also be capable of identifying which factors or latent variables are most important and should be included in the final model. Calibration models (i.e., models developed from a **training data set**) that are over-fit contain too many factors or latent variables and thus take into account random noise (Lupoi et al., 2014). Over-fit models may appear to be more accurate when tested against training data, but will make less accurate predictions when used on a **testing data set**, and can be avoided by using cross validation (see subsequent section) (Moore et al., 2010; Lupoi et al., 2014).

KEY CONCEPT 4. **Training and testing data sets**

Training data are used to build a predictive model, whereas testing data, which were not included in the training data set, are used for model validation and can also be used to assess model sensitivity and specificity.

**Figure 2:**
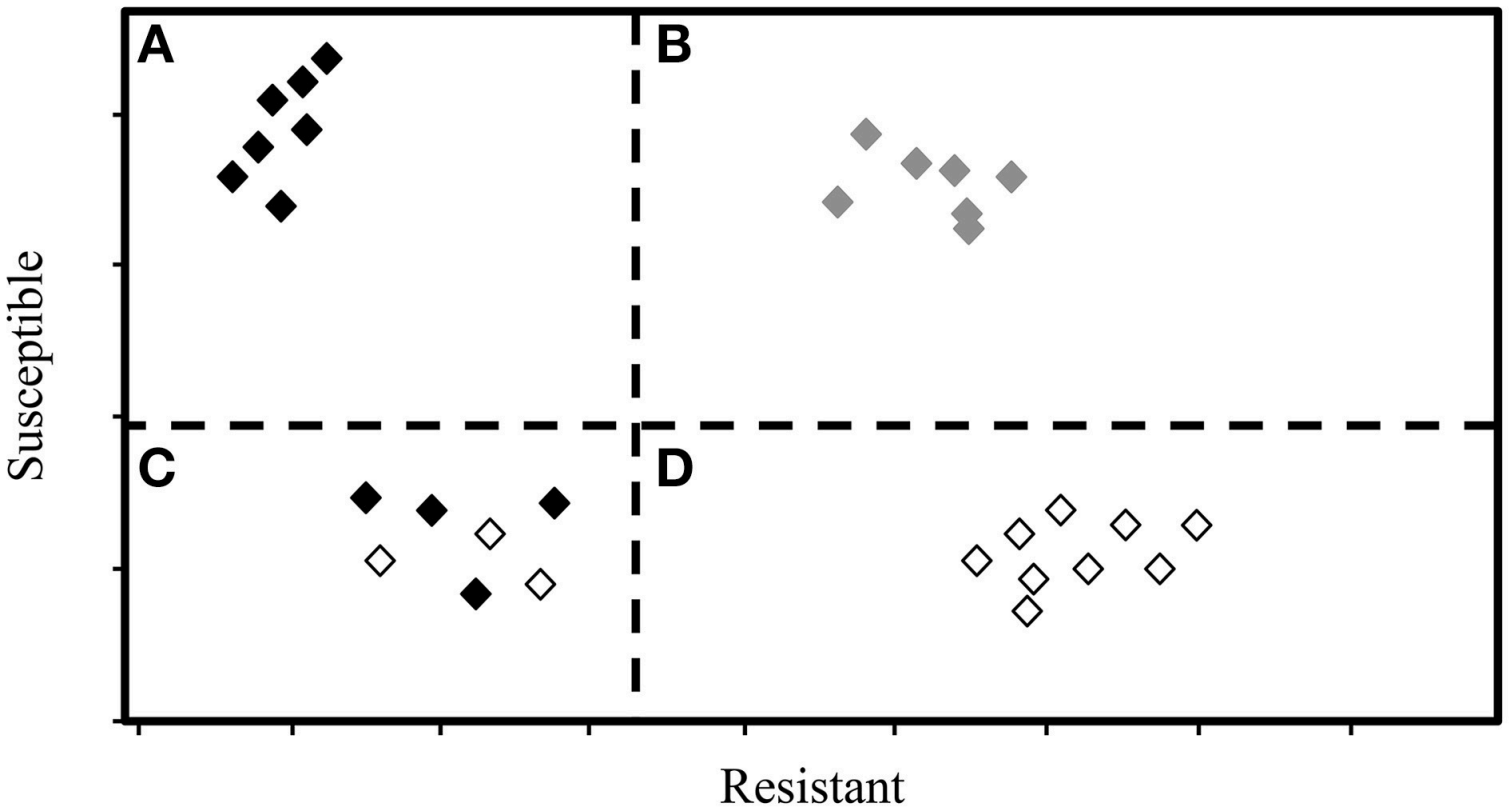
**This hypothetical output from SIMCA analysis displays the relative, dimension-free distance between samples (trees), and groupings of individual trees into resistant and susceptible phenotypes**. Dashed lines represent critical sample residual thresholds. Trees in quadrant **(A)** would be classified as resistant by the SIMCA model, while trees in quadrant **(B)** would be classified as neither resistant nor susceptible, i.e., as ambiguous. Trees in quadrant **(C)** could be classified as either resistant or susceptible, and may therefore include trees of intermediate phenotype. Trees in quadrant **(D)** would be classified as susceptible.

There are two primary types of chemometric models: supervised (*a priori* groupings used to inform the model) and unsupervised (*a priori* groupings are not used to inform the model). There are many methods for supervised analysis, including: SIMCA, partial least squares discriminant analysis (PLS-DA), PLSR, and linear discriminant analysis (LDA) (Sankaran et al., 2010; Guzmán et al., 2012; Durgante et al., 2013). For supervised analysis, samples must be grouped (i.e., phenotyped) using reliable and accurate methods. Among unsupervised approaches, PCA is used most widely. Based on our review of literature related to trees and IR and Raman spectroscopy, we compiled a list of commonly used chemometric methods and included a general description of each method in Table 2. Finally, while some methods can be used independently (e.g., SIMCA), others should be used in conjunction (e.g., PCA combined with DFA or LDA) to identify important spectra and to develop models capable of discriminating between groups of trees that vary in disease susceptibility. See Figure 2 for an example of a hypothetical output from SIMCA analysis.

**Table 2:** **List of commonly used chemometric methods for visualizing and mining spectral data, and for building predictive models from spectral data for trees**.

**Chemometric method**	**Description**
Discriminant function analysis (DFA)	Supervised projection method, which identifies regions that are important for separating groups. *A priori* groupings used to measure within and between group variance, and then used to define optimal function for discriminating *a priori* groups (Martín et al., 2008).
K-nearest neighbors (KNN)	Compares the distance between unknown samples (testing set) and samples in the training set. Samples are classified based on proximity to training set samples (Guzmán et al., 2012).
Linear discriminant analysis (LDA)	A supervised method for classifying data with two or more classes, which selects latent variables that maximize variance between groups and minimize variance within groups. Uses a discriminant function to assign classes to unknown samples (Sankaran et al., 2010; Durgante et al., 2013).
Principal components analysis (PCA)	Unsupervised method for visualizing and grouping data based on natural clustering patterns. Can be used to reduce the dimensionality of data, minimize co-linearity, examine spectral variance, and identify outliers (Gierlinger et al., 2003; Sankaran et al., 2010; Guzmán et al., 2012; Durgante et al., 2013; O'Reilly-Wapstra et al., 2013; Lupoi et al., 2014).
Partial least squares regression (PLSR)	Combines methods for reduction of high dimensional and potentially co-linear data with regression to develop predictive (calibration) models for quantitative traits of interest. This supervised method is commonly used for the analysis of NIR spectra (Fackler et al., 2007; Moore et al., 2010; Conrad et al., 2014; Warburton et al., 2014).
Partial least squares discriminant analysis (PLS-DA)	Supervised classification analysis that resolves separation between groups and identifies the most important variables for discriminating between groups. Similar to PLSR but with categorical (qualitative) response variables (Guzmán et al., 2012).
Soft independent modeling of class analogy (SIMCA)	A supervised classification method that develops principal components models for each training group and identifies important variables for discriminating between groups. Can be used to predict group memberships of unknown samples (Guzmán et al., 2012; Conrad et al., 2014).

*Please refer to cited papers for additional information*.

### Model validation

The final step in analysis of chemical fingerprint data, **model validation** is the most important for ensuring that accurate and good-fitting models have been developed (see Moore et al., 2010; Sankaran et al., 2010; Guzmán et al., 2012; O'Reilly-Wapstra et al., 2013; Lupoi et al., 2014). Validation can include testing the calibration model (i.e., the model derived from the training data set) on a testing data set, or using cross validation (Foley et al., 1998). For cross validation, the sample population is randomly divided into smaller groups and an iterative process is used to test predictions of one group based on calibration models developed from the remaining groups (Foley et al., 1998). Leave-one-out cross validation (LOOCV) is a commonly used method that involves removing one individual (instead of group) at a time (Durgante et al., 2013; Lupoi et al., 2014). Validation using a testing set can be used when the sample size is large enough to split data sets into two, while cross validation methods may be more appropriate for data sets with smaller samples sizes and are more convenient because a testing data set does not need to be maintained (Foley et al., 1998).

KEY CONCEPT 5. **Model validation**

In order to ensure accurate predictions, models must be validated by testing model predictions on a naïve data set (i.e., testing data set) or by using cross-validation methods, such as leave-one-out cross validation.

## Chemical fingerprinting of trees using IR and raman spectroscopy

### FT-IR spectroscopy

For more than 10 years, FT-IR spectroscopy combined with chemometrics has been used as a tool to discriminate between trees that differ in disease susceptibility before and after pathogen infection. The elm (*Ulmus* spp.)-*O. novo-ulmi* pathosystem (Martin et al., 2005a,b; Martín et al., 2007, 2008) is the most studied system. Using FT-IR spectroscopy combined with chemometrics (e.g., PCA and DFA), Martin et al. (2005a,b) were able to monitor chemical changes in resistant and susceptible *U. minor* following infection with *O. novo-ulmi*. Using the same approach, Martín et al. (2007) detected changes in chemical profiles, specifically in lignin levels, of inoculated *U. minor* and *U. minor* × *U. pumila* hybrids compared to their respective non-inoculated controls. Finally, they used chemical fingerprints from healthy xylem tissue to separate resistant *U. pumila*, susceptible *U. minor*, and resistant *U. minor* clones and identify spectral bands that were important for discriminating between those groups (Martín et al., 2008).

More recently, Hardoim et al. (2015) used FT-IR spectroscopy and chemometrics to examine changes in the metabolic patterns of *Quercus suber* roots following infection with the pathogen *Phytophthora cinnamomi*; significant differences in the intensity of certain spectral bands were detected between inoculated and mock-inoculated plants. Vivas et al. (2014) also recently used a combined FT-IR spectroscopic and chemometric approach to analyze maternal effects on the MIR spectrum of *Pinus pinaster* before and after inoculation with the pathogen *Fusarium circinatum*, the causal agent of pitch canker disease. They found that control seedlings from an unfavorable maternal environment, which were previously found to be less tolerant to *F. circinatum* (compared to seedlings from a favorable maternal environment), showed higher mean absorbances in the MIR spectral region compared to control seedlings from a favorable maternal environment; the same pattern held for seedlings after pathogen inoculation (Vivas et al., 2014). The authors concluded that variation in the intensity of the MIR spectrum may be associated with seedling carbohydrate content, and changes in carbohydrate content of seedlings following infection may impact their tolerance to *F. circinatum* (Vivas et al., 2014).

In our own work, we used a combined FT-IR spectroscopy and chemometric approach to distinguish between resistant and susceptible *Q. agrifolia* from natural populations before infection with the pathogen *P. ramorum* (Conrad et al., 2014). SIMCA analysis was used to discriminate between resistant and susceptible *Q. agrifolia* trees and to identify the most important spectra for discriminating between those groups (Conrad et al., 2014). In addition, PLSR was used to estimate the concentration of specific phenolic compounds previously associated with *Q. agrifolia* resistance to *P. ramorum*, based on FT-IR spectra.

### NIR spectroscopy

Even though NIR reflectance spectroscopy has been used widely, the technique has not been used extensively to screen trees for disease resistance. FT-NIR combined with PLSR was used to predict larch (*Larix* spp.) heartwood durability—a trait of interest in many tree breeding programs—and PCA was used to examine spectral variation between durability classes (Gierlinger et al., 2003). Furthermore, NIR spectroscopy combined with PLSR was used to predict decay resistance of *Pinus sylvestris* heartwood to the brown rot fungus *Poria placenta* (Flæte and Haartveit, 2004). Although the authors were primarily interested in using the technology to assess natural tree durability after harvesting, the results suggest that NIR spectroscopy can be used to detect chemical differences between groups that are phenotypically different (Flæte and Haartveit, 2004).

The ability to detect chemical differences between groups is important, because plant-derived chemicals are often associated with tree defense responses. For example Moore et al. (2010) used NIR reflectance spectroscopy to examine the relationship between palatability of *Eucalyptus* foliage and the total amount of formylated phloroglucinol compounds (FPCs). FPCs are known deterrents of koala feeding (Moore et al., 2010). These authors were able to estimate the total concentration of FPC in *Eucalyptus* foliage using calibration models that were created using NIR spectra and concentrations of FPC in *Eucalyptus* foliage quantitated by high performance liquid chromatography. Estimates were then combined with spatial tree distribution data to produce palatability maps based on koala feeding preferences of *Eucalyptus* foliage. A similar approach could be used to map the distribution of disease resistant trees on a landscape scale.

Most recently, O'Reilly-Wapstra et al. (2013) used chemical fingerprints generated from NIR reflectance spectroscopy (using a FT-NIR spectrometer equipped with a fiber optic probe), in combination with chemometric analysis (PCA and linear models), to differentiate between multiple generations of interspecific *Eucalyptus globulus* × *Eucalyptus nitens* hybrids and parent species. While the authors did not use this method to screen trees for disease resistance, they clearly showed that the technique can be used to discriminate between groups based on genetic differences in chemistry. Since tree disease resistance is genetically based and often an inherited trait, it is plausible that NIR spectroscopy and chemometrics could also be used to distinguish between genetic-based differences in resistance that manifest as differences in chemical composition. Further applications and examples of NIR spectroscopy for analyzing the chemical composition, anatomical features, mechanical properties, and other attributes of trees can be found in a recent review by Tsuchikawa and Kobori (2015).

### Raman spectroscopy

We were unable to find any papers where Raman spectroscopy and chemometrics were used to distinguish between trees that varied in disease susceptibility. However, it has been used to measure and estimate various chemical constituents of trees. For example, FT-Raman spectroscopy was used to study chemical changes in waterlogged *Pinus* spp. and *Quercus* spp. (Petrou et al., 2009). The authors assessed the depletion of cellulose, hemicellulose, and lignin in ancient wood samples from these tree species. Raman spectroscopy was also used to measure the lignin syringyl/guaiacyl (S/G) ratio of 17 eucalypt and *Acacia* tree species (Lupoi et al., 2014). Lupoi et al. (2014) were interested in assessing whether or not Raman (but also MIR and NIR) could be used to screen trees for biofuel feedstock candidates. While all three methods could be used to estimate S/G ratios, estimations were most accurate when determined using spectral data collected from Raman and MIR spectroscopy (Lupoi et al., 2014). Finally, a portable Raman spectrometer (equipped with a laser diode and optical fiber) was used to assess the quality of fruit from olive trees and could differentiate between sound (i.e., higher quality) and ground (i.e., lower quality) fruit using PCA, SIMCA, PLS-DA, and KNN (Guzmán et al., 2012).

Raman spectroscopy is clearly capable of detecting chemical differences between sample groups. Since plant-derived chemicals (e.g., phenolics) can be associated with tree resistance (reviewed in Witzell and Martin, 2008), this technology could also be used to identify spectra associated with variation in tree disease resistance.

## Future application of IR and raman spectroscopy for identifying disease resistant trees

In the preceding sections, we described methods for chemically fingerprinting trees using IR and Raman spectroscopy. Chemical fingerprinting, when combined with chemometrics, is a powerful tool that can be used to distinguish between groups of trees that vary in disease susceptibility. Alternatively, these methods could be used to estimate quantitative traits of interest, like the concentration of plant-derived chemicals, which may be associated with resistant tree responses.

Many of the studies referenced above utilized benchtop spectrometers for chemical fingerprinting of samples; however, there are many portable and handheld devices available that could be used directly in the forest to screen larger numbers of trees for disease resistance, once protocols to deal with fresh tissues have been optimized (see reviews by Sorak et al., 2012; Cozzolino, 2014). Handheld devices have not yet been used for this purpose, though on-site screening methods using NIR spectroscopy have been developed to predict sugarcane smut resistance for plant breeding (Purcell et al., 2011) and portable NIR spectrometers were used for in-forest prediction of Kraft pulp yield and cellulose content of standing trees (Meder et al., 2011). Although Meder et al. (2011) acknowledge that their approach needs to be modified to improve accuracy of model predictions, they believe hand-held devices are a feasible, rapid, and low-cost alternative that can be used to screen trees for breeding programs.

Handheld or automated laboratory devices will need to be utilized if chemical fingerprinting by IR and Raman spectroscopy is to be implemented on more high-throughput scales to efficiently screen trees for disease resistance. Instruments for these types of analyses are commercially available and chemometric pipelines for analyzing large data sets are well established. The next steps will be to scale-up existing experiments using handheld and portable devices, increase sample diversity within training and testing data sets, and validate predictive models for in-forest assessment of tree resistance. Once these steps have been completed, chemical fingerprinting and chemometrics should be viewed as a reliable method for screening individuals in tree breeding programs and for managing forest diseases by identifying and utilizing naturally resistant trees.

## Author contributions

AC and PB discussed content and structure of review prior to writing review. AC reviewed relevant literature and compiled initial draft of review. PB reviewed first and subsequent drafts and contributed content. AC revised manuscript based on PB contributions and feedback.

### Conflict of interest statement

The authors declare that the research was conducted in the absence of any commercial or financial relationships that could be construed as a potential conflict of interest.
